# The N‐terminal D1 domain of *Treponema pallidum *flagellin binding to TLR5 is required but not sufficient in activation of TLR5

**DOI:** 10.1111/jcmm.14617

**Published:** 2019-09-07

**Authors:** Man Xu, Yafeng Xie, Manyi Tan, Kang Zheng, Yongjian Xiao, Chuanhao Jiang, Feijun Zhao, Tiebing Zeng, Yimou Wu

**Affiliations:** ^1^ Institution of Pathogenic Biology, Medical College Hunan Provincial Key Laboratory for Special Pathogens Prevention and Control Hunan Province Cooperative Innovation Center for Molecular Target New Drug Study University of South China Hengyang China; ^2^ Department of Clinical Laboratory The Second Affiliated Hospital of University of South China Hengyang China; ^3^ Hunan Provincial Center for Disease Control and Prevention Changsha China; ^4^ Department of Laboratory Medicine, The Second Xiangya Hospital Central South University Changsha China

**Keywords:** flagellin, inflammation, N‐terminal D1 domain, TLR5 signalling, *Treponema pallidum*

## Abstract

Syphilis is a chronic bacterial infection caused by *Treponema pallidum* (*T pallidum*) and the pathogenesis that *T pallidum* infection induces immunopathological damages in skin and other tissues remains unclear. We have previously reported that recombinant flagellins of *T pallidum* can elicit IL‐6 and IL‐8 transcriptions via TLR5 pathway. To identify the domains which induced the pro‐inflammatory activity and the importance of the interactions between TLR5 and domains, homology‐based modelling and comparative structural analyses revealed that *Tp*flagellins can combine with TLR5 directly. Deletion mutations showed that the ND1 domain binding to TLR5 is required but not sufficient in TLR5 activation. Moreover, site‐directed mutagenesis analysis indicated that the arginine residue (*Tp*flagellins R89) of the ND1 domain and its adjacent residues (*Tp*flagellins L93 and E113) constitute a hot spot that elicits IL‐6, IL‐8 transcriptions and TLR5 activation, and affects the binding of *Tp*flagellins to TLR5. Taken together, these results give insight into the pathogenesis of *T pallidum* and may contribute to the future design of *Tp*flagellins‐based therapeutics and syphilis vaccine.

## 1 INTRODUCTION

*Treponema pallidum* subsp *pallidum* (*T pallidum*) is the causative agent of syphilis, a multistage sexually transmitted disease with an estimated prevalence of 36 million individuals affected worldwide and 12 million new cases per year.^1^ It is responsible for chronic infections in compromised individuals and disseminates throughout the entire body of patients through bloodstream, therefore leading to diverse clinical manifestations.^2^ In spite of the great progress in the diagnosis of syphilis and long‐term in vitro culture of the *T pallidum*,^3^, ^4^ few studies were performed on the pathogenesis of *T pallidum*.^5^, ^6^, ^7^ Unlike other external flagellated bacteria, *T pallidum*, an obligate microaerophilic bacterial pathogen,^8^ is motile via the endoflagella that undulating their bodies as travelling planar waves, thereby driving its movement to swim through viscous fluids, such as blood and interstitial fluid.^9^, ^10^ The flagellar filament of *T pallidum* is composed of three core protein (FlaB1, FlaB2 and FlaB3) and two outer layer proteins (FlaA1 and FlaA2).^11^ The role of flagellar antigen‐induced immune stimulation in the virulence of many pathogenic microorganisms, including *Salmonella typhimurium* and *P aeruginosa*, has been well established.^12^, ^13^, ^14^

Flagellin, the major structural protein of bacterial flagella, can promote pathogens to adhere and invade into host cells as a virulence factor^15^ and activate innate immunity through the TLR5 and NAIP5/NLRC4 pathway,^16^, ^17^, ^18^, ^19^ which will contribute to the immediate clearance of pathogens from the host. The flagellin molecule contains two to four domains, D0‐D3. Of these, the D0 and D1 domains (N/C regions) are highly conserved between organisms, which are crucial for the immunostimulatory activity of flagellin.^17^, ^20^, ^21^ However, the D2 or D3 domain (middle hypervariable region), which is essential for flagellin antigenicity^22^ and could cause the undesirable toxicity of flagellin‐based therapeutics,^23^ has vast diversity in their sequence, size and composition in various bacterial strains and is even absent in some bacterial species, including *T pallidum*. Since flagellin was considered as an activator in shaping both the innate and adaptive arms of immunity in the first line of defence against flagellated pathogenic bacteria, it has been shown to enhance cross‐protective response of related antigens against diverse infectious diseases as a vaccine carrier protein or a vaccine adjuvant, and some of the vaccines eventually made their way into human clinical trials.^24^, ^25^, ^26^ More significantly, flagellin‐activated TLR5 has also exhibited anti‐tumour, anti‐metastatic immunotherapy and radioprotective property, and has shown tremendous potential in resisting cancer cell growth and radiation‐associated gastrointestinal tissue damage.^23^, ^27^, ^28^, ^29^

TLR5, consisting of an extracellular domain with leucine‐rich repeats (LRRs), a transmembrane domain, and an intracellular Toll/IL‐1 receptor (TIR) domain, is a member of evolutionarily conserved type I transmembrane receptors located on the cell surface that provides a vital link between innate and adaptive immunity.^30^ It has been reported that TLR5 could present on a variety of cells, including monocytes, macrophages, basophils, neutrophils, lymphocytes, NK cells, dendritic cells (DCs), endothelial cells and epithelial cells.^31^, ^32^, ^33^ TLR5 recognizes flagellin with the extracellular domain and recruits downstream adaptor molecules binding to the intracellular TIR domain, which will activate the MAPK and NF‐κB signal pathway and further lead to the expression of a variety of gene, including pro‐inflammatory cytokines, chemokines, nitric oxide (NO) and host‐defence proteins.^34^, ^35^

Structural study has revealed that TLR5‐flagellin interaction mediated through two distinct binding sites. The first facilitates formation of a 1:1 TLR5‐flagellin complex, and the second guides the formation of a 2:2 complex necessary for signalling.^36^ It has been identified that flagellins from Gram‐negative *Salmonella enterica subspecies enterica serovar Dublin*, *P aeruginosa* and Gram‐positive *Bacillus subtilis* are all binding to the TLR5 ectodomain with amino acid residues within the conserved D1 domain.^36^, ^37^, ^38^ However, as a result of the variations in the sequences and domains of flagellins, flagellins from diverse bacterial species use the unequal TLR5‐recognition mechanism. For instance, previous reports in FliC flagellin from *Salmonella enterica serovar Enteritidis*, *S enterica serovar Typhimurium* and *Pseudomonas aeruginosa* have shown that the hypervariable region is essential for the activation of TLR5.^39^ In addition, some researchers found that the D1 domain of flagellins is essential for the activation of TLR5 in mammals.^38^, ^40^, ^41^ However, a recent study revealed that the C‐terminal D0 domain plays a crucial role in TLR5 activation.^42^ Despite our knowledge of other flagellins and TLR5 binding interface based on the crystal structure, the contribution of each flagellin residue in flagellin‐TLR5 binding and TLR5 activation has not been elucidated and therefore there is an urgent need for us to investigate the interactions between *T pallidum* flagellins (*Tp*flagellins) and TLR5.

We previously showed that *Tp*flagellins are capable of inducing IL‐6 and IL‐8 productions through TLR5‐dependent MAPK and NF‐κB signalling^43^ and inhibiting the dissemination of *T pallidum* in rabbits.^26^ The purpose of the present investigation was to further define the regions and the specific amino acid residues of *T pallidum* flagellins in receptor activation and pro‐inflammatory activity, which will contribute to establishing the foundation work for the future design of *Tp*flagellins‐based syphilis vaccine and therapeutics.

## 2 MATERIALS AND METHODS

### 2.1 Cell cultures

The human monocytic leukaemia cell line, THP‐1 (American Type Culture Collection) was cultured in complete medium RPMI 1640 (Gibco, NY, USA), containing 10% foetal bovine serum (Gibico) supplemented with 100 U/mL penicillin and 100 mg/mL streptomycin at 37°C in 5% CO_2_. For experimental purposes, cells were resuspended in serum‐free medium and then seeded at 1 × 10^6^ cells/well in 6‐well tissue culture plates and then cultivated overnight.

### 2.2 Construction of wild‐type and mutant flagellin plasmid DNAs

The construction of a bacterial expression plasmid pET28a‐FlaB containing *T pallidum* flagellins‐coding sequences has been described previously.^44^ These plasmids were used as the template for subsequent mutagenesis. Deletion mutant plasmids were generated by one‐step PCR and cloned into the expression vector pET28a via restriction sites to give expression of N‐terminal His‐tag proteins. The chimeric NC without the hypervariable region was constructed using overlap extension PCR.^45^ Point mutations in the flagellin gene were created by circular PCR with DpnI digestion to eliminate background wild‐type plasmid. All primers used are given in Tables S1 and S2. All mutants were confirmed by DNA sequence analysis.

### 2.3 Expression and purification of mutant flagellins

*E coli Rosseta* (DE3) containing either wild‐type or mutant expression plasmids were grown at 37°C in Luria‐Bertani medium with kanamycin (50 mg/mL). Log‐phase cultures were induced with 0.5 mmol/L isopropyl‐β‐D‐thiogalactopyranoside (IPTG) for 6 hours at 30°C and then pelleted by centrifugation. The 6× His FlaB and all mutant flagellins were purified from inclusion bodies under denaturing conditions and then refolded. Protein concentrations were determined by using the BCA protein assay kit (Pierce, Rockford, IL, USA), and purity was verified by means of 15% sodium dodecyl sulphate‐polyacrylamide gel electrophoresis (SDS‐PAGE) and Coomassie blue staining. The LPS in each preparation of purified protein was removed by polymyxin B chromatography (Detoxi‐Gel; Pierce), and no detectable LPS were confirmed by Limulus assay (Chinese Horseshoe Crab Reagent Manufactory, Ltd., Xiamen, China). The samples were subpackaged and stored in aliquots at −80°C until needed.

### 2.4 Transient transfection

THP‐1 cells (1 × 10^6^ cells/well) were transfected with 4 μg interfering plasmids (psiRNA‐hTLR5 and control plasmids psiRNA‐LucGL3) using 10 μL Lipofectamine 2000 (Invitrogen, Carlsbad, USA) for 28 hours according to the manufacturer's instruction. After 28 hours of transfection, the cells were treated with 1 μg/mL FlaB1 mutant flagellins, 10 μg/mL FlaB2 mutant flagellins or 5 μg/mL FlaB3 mutant flagellins for selected time for further experiments.

### 2.5 qRT‐PCR

Total RNA was extracted from human THP‐1 cells using TRIzol Reagent (Invitrogen, Carlsbad, CA), the first‐strand cDNA synthesis using FastKing RT kit（KR116）(Qiagen, Shanghai, China) with random primers for quantitative reverse transcription PCR (qRT‐PCR) analysis according to the manufacturer's instruction. qRT‐PCR was performed with QuantiFast SYBR one‐step RT‐PCR (Qiagen, Shanghai, China) and LightCycler® 96 instrument (Roche Diagnostics, Roche Instrument Center AG, Rotkreuz, Switzerland). Primer sequences used in qRT‐PCR were as follows: GAPDH‐F (CAGGAGGCATTGCTGATGAT) and GAPDH‐R (GAAGGCTGGGGCTCATTT); IL‐6‐F (TACATCCTCGACGGCATCTC) and IL‐6‐R (TTTCAGCCATCTTTGGAAGG); IL‐8‐F (AGCTCTGTGTGAAGGTGCAGT) and IL‐8‐R (AATTTCTGTGTTGGCGCAGT). The PCR conditions were as follows: one cycle of initial denaturation (5 minutes at 95°C) and 40 amplification cycles (10 seconds at 94°C, 20 seconds at 58°C and 10 seconds at 72°C). Each RNA sample was performed in triplicate. The results normalized by using the reference gene GAPDH. Normalized relative quantities were calculated using the comparative threshold cycle (2^–△△Ct^) method.

### 2.6 Western blotting

THP‐1 cells, grown in 6‐well plates at a density of 5‐7 × 10^6^ cells per well, were stimulated with purified full‐length *T pallidum* flagellins and mutant flagellins for selected times. The cells then were lysed in lysis buffer (Beyotime Institute of Biotechnology, Jiangsu, China) containing a cocktail of protease inhibitor and phosphatase inhibitor for 60 minutes after being washed twice with ice‐cold phosphate‐buffered saline (PBS). Cell debris was removed by centrifugation at 4℃ with 10 000 × g for 15 minutes. The amount of protein in each sample was determined using the BCA assay. Further, the equal amounts of total protein were separated by 12% SDS‐PAGE gels and transferred to a PVDF membrane (Millipore, Bedford, MA, USA). The membranes were washed (1 × TBS buffer) and blocked with 5% nonfat milk in 1 × TBST (25 mmol/L Tris‐HCl [pH7.4], 137 mmol/L NaCl, 2.68 mmol/L KCl and 0.05% Tween 20) for 2 hours at room temperature. Then, the membranes were incubated with specific primary antibodies overnight at 4°C. Blots were washed three times for 15 minutes each with 1 × TBST buffer, followed by the addition of second antibodies for another 1.5 hours at 37°C. The antigen‐antibody complexes were detected with ECL reagent by Western blotting system G:BOX Chemi XXX9 (Syngene, Cambridge, UK).

### 2.7 Co‐immunoprecipitation binding assay

For direct co‐immunoprecipitation analysis studies, equal amounts of full‐length or mutant flagellin were incubated with soluble hTLR5 extracted with lysis buffer from THP‐1 cells (0.5 mL/100‐mm dishes) or incubated with lysis buffer as a control, then incubated with anti‐hTLR5 monoclonal antibody (mAb; Abcam, Cambridge, UK) (TLR5: TLR5 antibody (19D759.2)) at 4°C for 16 hours. The complexes were then incubated with 40 μL of Pierce agarose (cross‐linked 4% beaded agarose) for 1 hour at room temperature and washed 5 times with wash buffer (1× PBS, 0.02% Tween 20, pH 7.5), according to the manufacturer's protocol (Thermo Fisher Scientific). Then, they were eluted by boiling in 0.1% SDS at 95°C for 8 minutes. Immune complexes were separated by SDS‐PAGE and subjected to Western blotting analysis. The flagellin in the samples was detected with anti‐His antibodies.

### 2.8 Flagellin sequence alignments

Flagellin sequences were aligned by using ClustalW (http://www.genome.jp/tools-bin/clustalw). A phylogenetic tree was displayed with DNAMAN 6.0 program ( Lynnon Biosoft).

### 2.9 3D structure prediction

The 3D models of the *Tp*flagellins were predicted by Phyre 2.^46^ The structure comparisons of various flagellins were performed by using UCSF Chimera 1.12 program.^47^

### 2.10 Statistical analysis

Results are expressed as means ± SD of three experiments. GraphPad Prism 6.0 (GraphPad Software, Inc, La Jolla, CA) was used for statistics. Student's *t* test was used for statistical comparison of the data. Differences were considered statistically significant when the *P* value was less than .05.

## 3 RESULTS

### 3.1 Modelling of *Tp*flagellins structures and structural comparison of various flagellins

**Figure 1:**
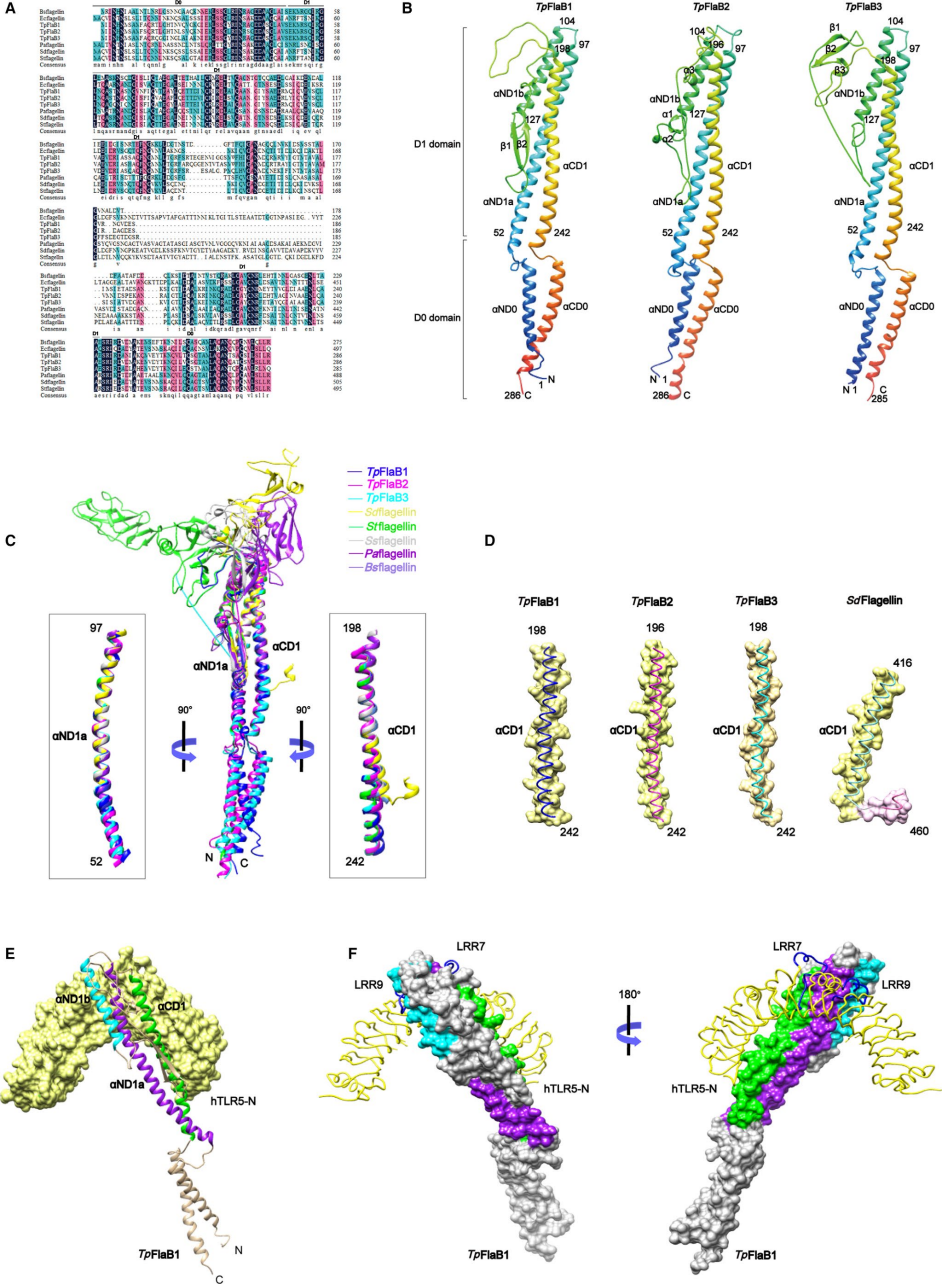
Modelling of *Tp*flagellins structures and structural comparison of various flagellins. A, Alignments of protein sequence from various flagellins. Protein sequences were retrieved from the Swiss‐Prot Database and aligned by ClustalW. Shading indicates areas where there is high conservation (dark) and moderate conservation (magenta and blue). *Bs*flagellin, *Bacillus subtilis* flagellin; *Ec*flagellin, *Escherichia coli* flagellin; *Pa*flagellin, *Pseudomonas aeruginosa*; *St*flagellin, *Salmonella typhimurium* flagellin; *Sd*flagellin, *Salmonella enterica subspecies enterica serovar Dublin* flagellin. B, Homology‐based structural model of *Tp*flagellins. The *Tp*flagellins structures are shown in rainbow‐coloured ribbons. Secondary structural elements are indicated. N, N‐terminus; C, C‐terminus. C, Overlays of the *Tp*FlaB1 structure (blue ribbon), *Tp*FlaB2 structure (magenta ribbon), *Tp*FlaB3 structure (cyan ribbon) and other flagellin structures (coils) are shown [*Sd*flagellin (PDB ID 3V47, yellow), *St*flagellin (PDB ID 1UCU, green), *Ss*flagellin (PDB ID 2ZBI, light grey), *Pa*flagellin (PDB ID 4NX9, purple) and *Bs*flagellin (PDB ID 5GY2, cornflower blue)]. The αND1a and αCD1 regions of *Tp*flagellins adopted different conformations from various flagellins and are shown in ribbons in the left and right boxes, respectively. D, The αCD1 helices of *Tp*flagellins and *Sd*flagellin are shown as surface presentation and coils. E, Interactions of the *Tp*FlaB1 with the hTLR5 chain. *Tp*FlaB1 is shown in ribbons and hTLR5 chain is shown with yellow surface. F, Interactions of the *Tp*FlaB1 with the hTLR5 chain. *Tp*FlaB1 residues involved in hTLR5 binding are coloured by purple (αND1a), cyan (αND1b) and green (αCD1), hTLR5 chain is shown as yellow lines. The TLR5‐specific loops at LRR7 and LRR9 are shown as blue lines

Since our earlier results have shown that *T pallidum flagellins* FlaB1‐3 can up‐regulate IL‐6 and IL‐8 expressions via TLR5 pathway,^43^ the possibility of *Tp*flagellins in complex with TLR5 was analysed by bioinformatics software to confirm whether *Tp*flagellins‐mediated TLR5 activation occurs through a direct interaction between *Tp*flagellins and TLR5. As shown in Figure 1A, *Tp*flagellins and other TLR5‐activated flagellins were similar in amino acid sequence (identity, ~60%) in conserved D0 and D1 domains. Then, the 3D models of *Tp*flagellins were predicted by using homology modelling (Figure 1B). The structural comparison indicated that all of the three *Tp*flagellins contained the common three α‐helices (αND1a, αND1b and αCD1) in the D1 domain, which was shown to combine directly with TLR5 in an earlier reported *Sd*Flg‐zfTLR5 structure.^36^ However, the structure of three *Tp*flagellins was distinguished in residues 127‐198 where *Tp*FlaB1 contained two β‐hairpin (Figure 1B, left), *Tp*FlaB2 contains three α‐helices (Figure 1B, middle), and *Tp*FlaB3 contains three β‐hairpin (Figure 1B, right).

*Tp*flagellins were structurally similar to its orthologs (*St*flagellin, *Pa*flagellin, *Ss*flagellin, *Sd*flagellin and *Bs*flagellin) in that they contained three α‐helices bundle structure (Figure 1B,C).^36^, ^37^, ^38^, ^48^, ^49^, ^50^ The *Tp*flagellin structures were the same as the majority of flagellins except for *Sd*flagellin, which contained two helices in the C‐terminal of the D1 domain (CD1 domain) (Figure 1C,D); however, the CD1 domain in the BcFlg‐hTLR5 complex structure was also discontinued to two α‐helices.^51^ This comparative analysis of various flagellin structures suggested that the CD1 domain was structurally diverse. To further ensure that the *Tp*flagellins were able to combine with TLR5, the structural analysis of a complex formation between *Tp*FlaB1 and human TLR5 (hTLR5) was performed in silico (Figure 1E,F). It was consistent with previous reports that *Tp*FlaB1 formed a 1:1 complex with TLR5 through three α‐helices (αND1a, αND1b and αCD1) in D1 domain.

### 3.2 Pro‐inflammatory mediators induced by the deletion mutant flagellins

**Figure 2:**
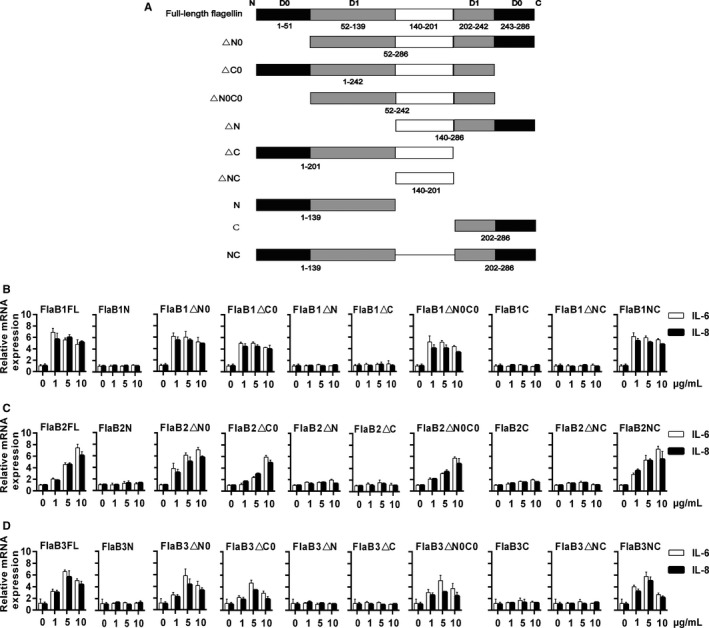
The deletion mutant flagellins induce pro‐inflammatory mediators. A, Wild‐type and deletion mutant forms of *Tp*flagellins are represented schematically. Wild‐type *Treponema pallidum* protein consists of 286 amino acids and three functional domains. Amino acids 1‐139 represents N‐terminus, 202‐286 represents C‐terminus, and 140‐201 represents hypervariable region of flagellins. THP‐1 cells were stimulated with 1‐10 μg/mL FlaB1 (B) [FlaB2 (C) or FlaB3 (D)] mutant flagellins for 24 h. The gene transcription levels of IL‐6 and IL‐8 were analysed by qRT‐PCR. Data are presented as mean ± SD of three independent experiments

To begin the molecular analysis of mutant *Tp*flagellins, we first made truncated recombinantly expressed proteins with deletions in the N‐terminus (△N, 139‐286; △N0, 52‐286), C‐terminus (△C, 1‐201; △C0, 1‐242), hypervariable region (NC, 1‐138 + 202‐286) and N + C terminus (△NC, 139‐201; △N0C0, 52‐242; C, 202‐286; N, 1‐51) (Figure 2A). These mutant genes were successfully amplified by PCR (Figure S1A‐C) with appropriate primers (Table S1) and confirmed by DNA sequence analysis, and then, they were cloned into expression vector pET28a. These recombinant mutant proteins were expressed as His‐tagged proteins in *E coli Rosseta* (DE3) and purified as described under ‘MATERIALS AND METHODS’ (Figure S1D‐F). To identify the function of the conserved regions of *Tp*flagellins in eliciting pro‐inflammatory mediators, THP‐1 cells were stimulated with 1‐10 μg/mL FlaB1 (FlaB2 or FlaB3) mutant flagellins. As shown in Figure 2B‐D, recombinant flagellins N, △N, △NC, △C and C failed to induce IL‐6 and IL‐8 transcriptions; however, the recombinant NC induced significant transcriptions of IL‐6 and IL‐8 comparable with that of full‐length recombinant flagellins, and these indicated that the conserved D1 domain was essential for pro‐inflammatory mediators’ productions. Moreover, the recombinant △N0, △C0 and △N0C0 mutants lacking the whole or part of the D0 domain had no effect on pro‐inflammatory responses, which further implied that the conserved D1 domain of *Tp*flagellins was functionally important for pro‐inflammatory signalling in THP‐1 cells.

### 3.3 Mutant flagellins containing only D1 domains activate MAPK and NF‐κB

**Figure 3:**
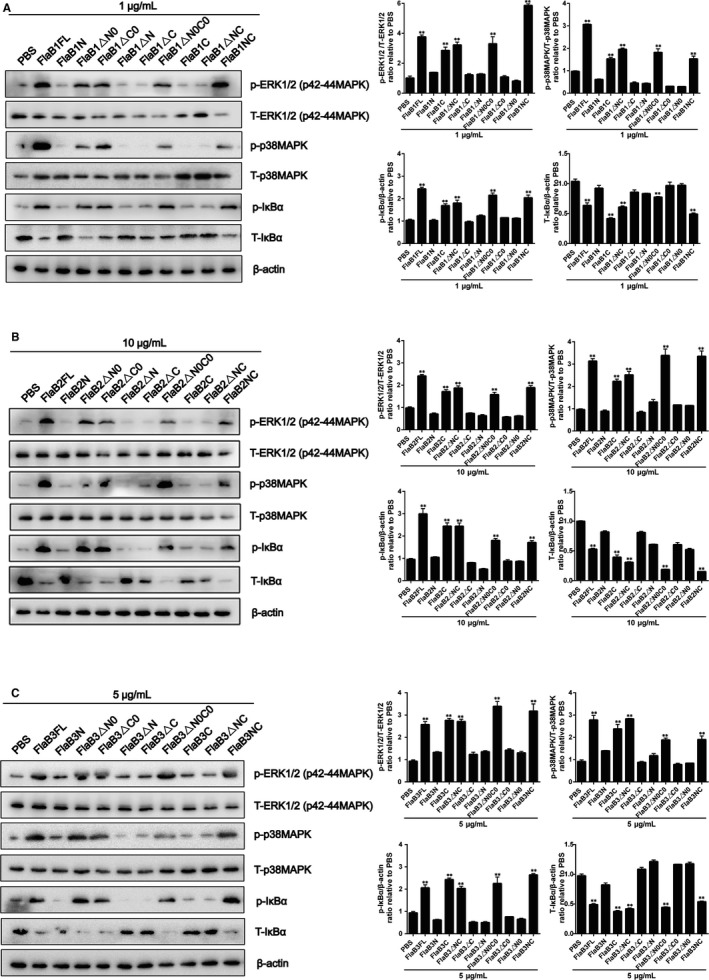
Mutant flagellins containing only D1 domains activate MAPK and NF‐κB. THP‐1 cells were treated with 1 μg/mL FlaB1 mutant flagellins (A), 10 μg/mL FlaB2 mutant flagellins (B) or 5 μg/mL FlaB3 mutant flagellins (C) for 1 h, and then, the total protein was extracted. The expression of nonphosphorylated and phosphorylated forms of ERK1/2 (p42‐44MAPK), p38MAPK and IκBα were analysed by Western blotting. ERK1/2 (p42‐44MAPK), p38MAPK and IκBα phosphorylation were quantified by ImageJ software and normalized with total protein or β‐actin. Data are presented as mean ± SD of three independent experiments. **P < .01 versus PBS control group

Because our previous studies have shown that MAPK and NF‐κB pathways can mediate *Tp*flagellins‐induced IL‐6 and IL‐8 expressions,^43^ Western blotting was used to investigate flagellins interaction with THP‐1 cells in this study. After THP‐1 cells were stimulated with 1 μg/mL FlaB1 (10 μg/mL FlaB2 or 5 μg/mL FlaB3) mutants, it can be observed in Figure 3A‐C that the recombinant *Tp*flagellin NC, △C0, △N0 and △N0C0 were able to activate the ERK1/2 (p42‐44MAPK), p38MAPK and IκBα in THP‐1 cells as well as the full‐length flagellins, whereas mutants N, △N, △NC, △C and C exhibited activity similar to that of untreated cells. These findings supported our earlier conclusion that the conserved D1 domain of *Tp*flagellins was essential for pro‐inflammatory activity.

### 3.4 Mutant flagellins containing only ND1 domains bind to human TLR5

**Figure 4:**
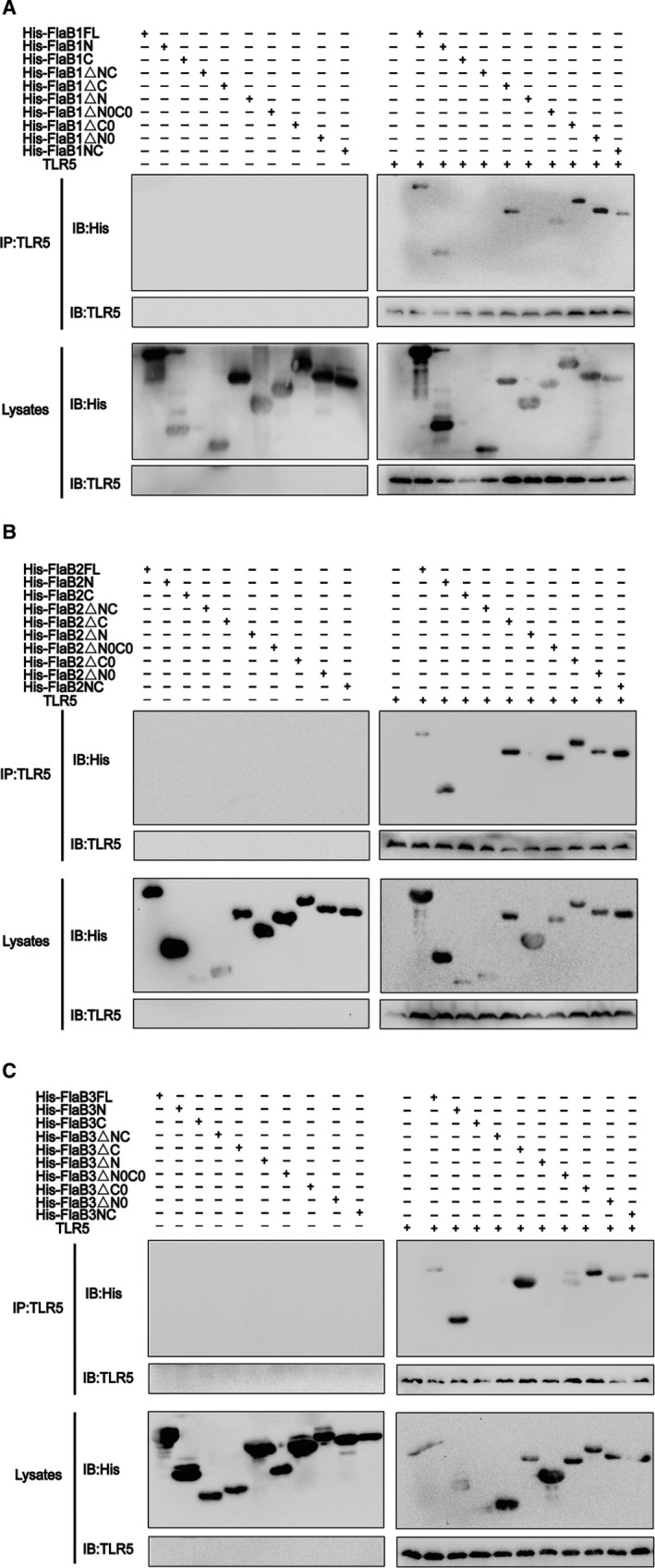
Mutant flagellins containing only ND1 domains bind to human TLR5. (A‐C) Co‐immunoprecipitation was performed to assess the binding of the mutated proteins to human TLR5. 30 μg of each protein was added to lysed THP‐1 cells (or lysis), then incubated with anti‐hTLR5 antibody at 4°C for 16 h, half of the lysates were incubated with 40 μL of beads for 1 h at room temperature and washed 5 times with wash buffer, and then, they were eluted and boiled for Western blotting analysis; the other half of the lysates were boiled and separated by SDS‐PAGE for Western blotting analysis. TLR5 polyclonal antibody was used for the detection of TLR5. Anti‐His antibodies were used for the detection of wild‐type flagellin and mutants

The previously reported crystal structure of zebrafish TLR5 in complex with the *Salmonella* flagellin FliC visualized the D1 domain is a conserved TLR5 binding hot spot.^36^ To confirm that the *Tp*flagellins‐mediated TLR5 activation occurs through a direct interaction between flagellin and TLR5, and to examine whether *Tp*flagellins induce TLR5 activation through the common binding site with other species of flagellins, we used co‐immunoprecipitation to detect the combination between mutant flagellins and human TLR5. As shown in Figure 4A‐C, mutant flagellins containing ND1 domains (NC, △C, △C0, N, △N0 and △N0C0) successfully binded to the TLR5, while the truncated flagellins lacking of ND1 domains (△N, △NC and C) affected the binding efficiency of flagellin molecule to TLR5. This was in agreement with previous structural studies where the primary binding site of flagellin to TLR5 was located within the D1 region,^36^ while inconsistent with the results of the previous TLR5 activation and IL‐6 and IL‐8 transcriptions. These results suggested that the role of the *Tp*flagellins in TLR5 activation was largely required for the binding of conserved N‐terminal D1 region to human TLR5.

### 3.5 Identification of the D1 domain of *Tp*flagellins that affect TLR5 signalling

**Figure 5:**
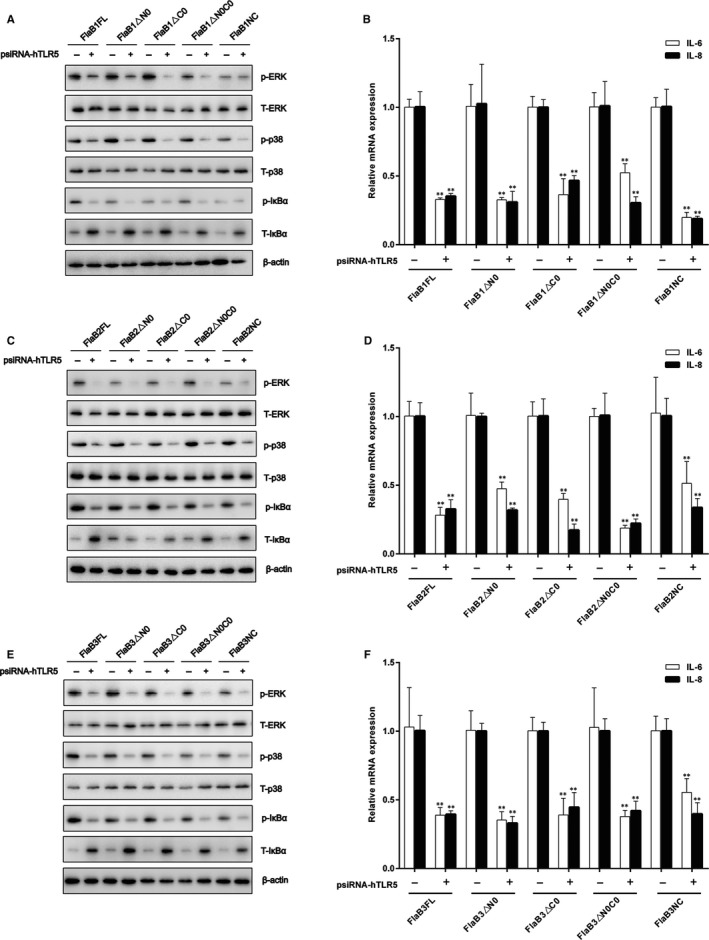
Identification of the D1 domain of *Tp*flagellins that affect TLR5 signalling. THP‐1 cells were transfected with psiRNA‐hTLR5 (psiRNA‐LucGL3 was used as a control) for 28 h, then stimulated with 1 μg/mL FlaB1 mutant flagellins (A), 10 μg/mL FlaB2 mutant flagellins (B), or 5 μg/mL FlaB3 mutant flagellins (C) for 1 h to analyse the expressions of nonphosphorylated and phosphorylated forms of ERK1/2 (p42‐44MAPK), p38MAPK, and IκBα, stimulated with 1 μg/mL FlaB1 mutant flagellins (D), 10 μg/mL FlaB2 mutant flagellins (E), or 5 μg/mL FlaB3 mutant flagellins (F) for 24 h to measure the transcription levels of IL‐6 and IL‐8 mRNA in the cells by using qRT‐PCR. Data are presented as mean ± SD of three independent experiments. *P < .05, **P < .01 versus corresponding control

To further identify the function of the conserved D1 domain of *Tp*flagellins in TLR5 activation, we used mutant flagellins containing D1 domains (NC, △C0, △N0 and △N0C0) to stimulate THP‐1 cells treated with TLR5 siRNA previously and full‐length flagellins as a positive control. Then, the activation of downstream signalling molecule ERK1/2 (p42‐44MAPK), p38MAPK, IκBα in THP‐1 cells, and the transcriptions of pro‐inflammatory cytokine IL‐6 and IL‐8 were monitored. As shown in Figure 5A‐F, all mutant flagellins containing D1 domains led to significant down‐regulations of the transcriptions of IL‐6 and IL‐8 after the silencing of TLR5, which was consistent with the activation of ERK1/2 (p42‐44MAPK), p38MAPK and IκBα. Taken together, these results indicated that the regions in flagellin required for pro‐inflammatory activity were also required for its functional activation of the TLR5 receptor.

### 3.6 Identification of residues in the N‐terminal D1 domain of *Tp*flagellins that affect inflammation response

**Figure 6:**
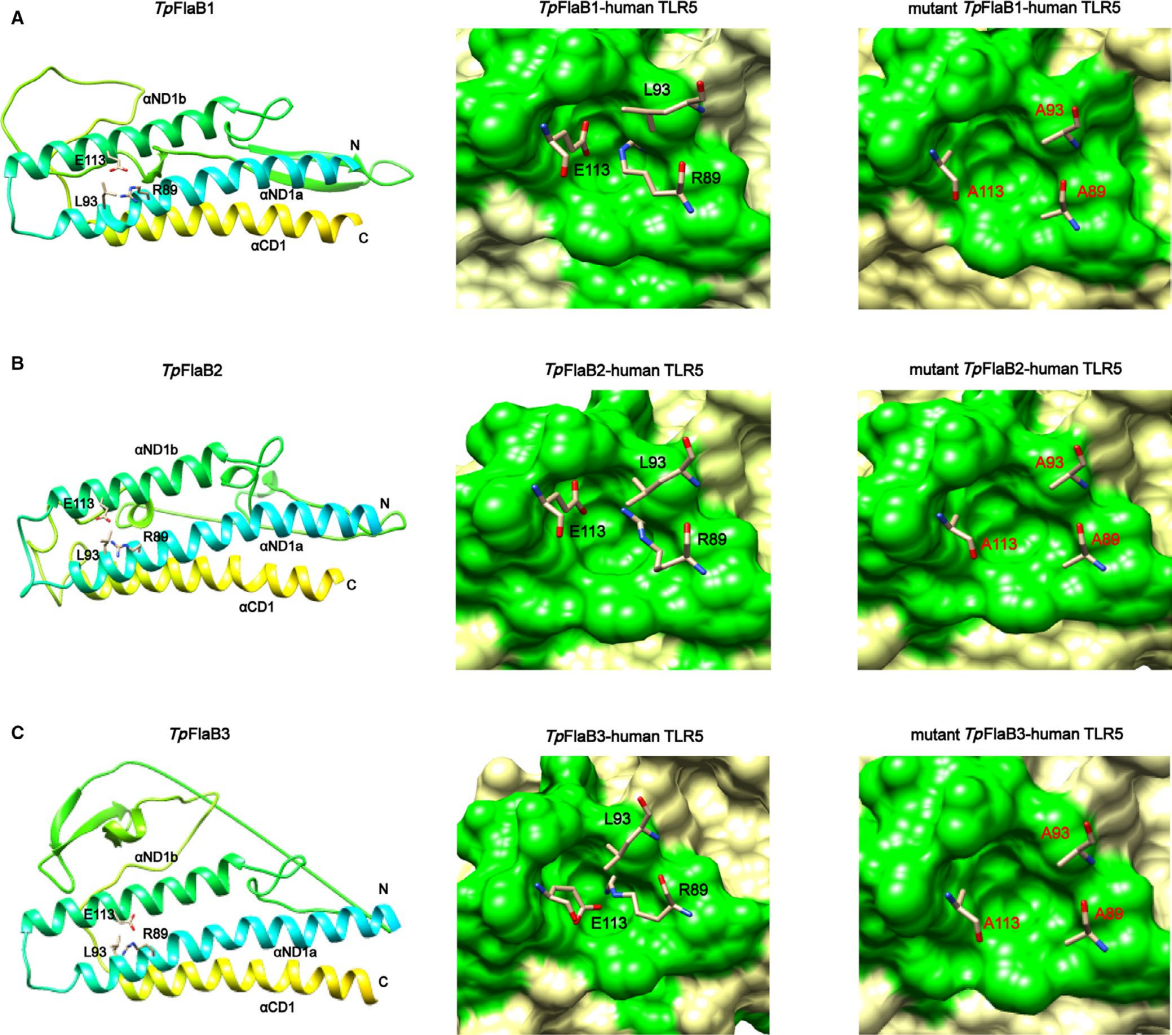
Computational model structures of wild‐type *Tp*flagellins and *Tp*flagellins mutants. Residues Arg89, Leu93 and Glu113 of *Tp*FlaB1 (A), *Tp*FlaB2 (B) or *Tp*FlaB3 (C) selected for alanine mutagenesis are present in wild‐type *Tp*flagellins structures (left). Residues Arg89, Leu93 and Glu113 of *Tp*FlaB1 (A), *Tp*FlaB2 (B) or *Tp*FlaB3 (C) observed in the cavity formed by the human TLR5 LRR9 loop (green surface) in the *Tp*flagellin‐human TLR5 model (middle). Residues Alanine 89, Alanine 93 and Alanine 113 of *Tp*FlaB1 (A), *Tp*FlaB2 (B) or *Tp*FlaB3 (C) observed in the cavity formed by the human TLR5 LRR9 loop (green surface) in the *Tp*flagellin‐human TLR5 model (right)

**Figure 7:**
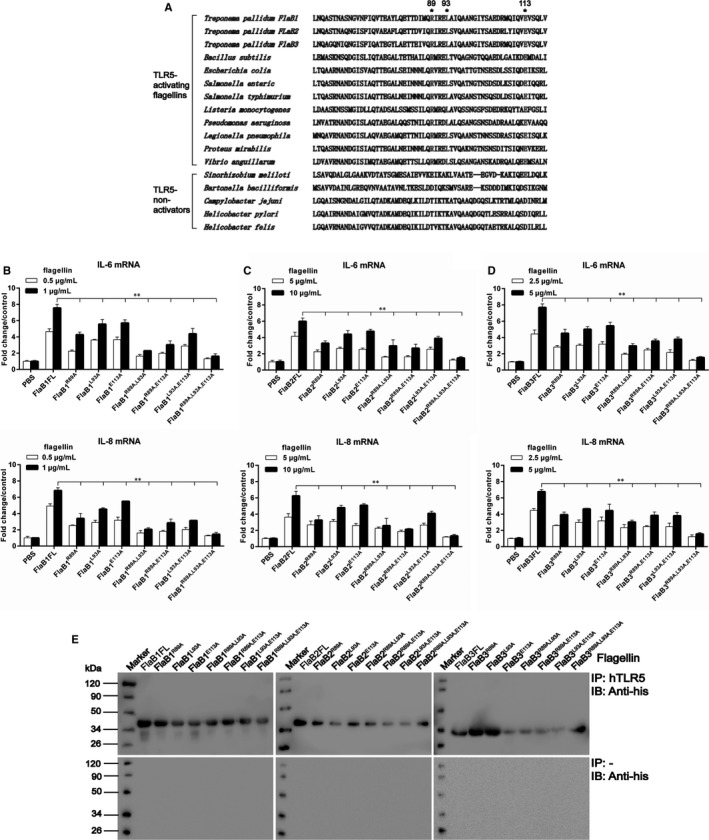
Identification of residues in the N‐terminal D1 domain of *Tp*flagellins that affect inflammation response. A, Amino acid alignment of ND1 domain flagellin sequences of several bacterial species and amino acid residues selected for alanine mutagenesis. B‐D, THP‐1 cells were stimulated with 0.5‐1 μg/mL FlaB1 mutant flagellins (B), 5‐10 μg/mL FlaB2 mutant flagellins (C) or 2.5‐5 μg/mL FlaB3 mutant flagellins (D) for 24 h. The gene transcription levels of IL‐6 and IL‐8 were analysed by qRT‐PCR. Data are presented as mean ± SD of three independent experiments. *P < .05, **P < .01 as compared with wild‐type flagellins. E, Co‐immunoprecipitation was performed to assess the binding of the mutated proteins to human TLR5. 30 μg of each protein was added to lysed THP‐1 cells (top) or control lysis (bottom) and then incubated with anti‐hTLR5 antibody at 4°C for 16 h, and the complexes were then incubated with 40 μL of beads for 1 h at room temperature and washed 5 times with wash buffer and eluted for Western blotting analysis. Anti‐His antibodies were used for the detection of wild‐type flagellin and mutants

Considering the fact that the D1 domain in N‐terminal was important for the pro‐inflammatory activity of *Tp*flagellins, we next sought to make clear the contribution of individual amino acids in inducing pro‐inflammatory response. Previous studies have suggested that the TLR5 LRR9 loop residues generate a cavity that accommodates the residues R89, L93 and E113 of flagellins through conserved key interactions.^36^, ^38^ The comparative structural analyses revealed that *Tp*flagellins possessed a common conserved molecular pattern for the TLR5 interaction with *Bs*flagellin (Figure 1C). Thus, we generated three different site‐specific mutations, single, double or triple (corresponding primers were listed in Table S2) in ND1 domain whereby arginine (Arg89) was changed to alanine (R89A), leucine (Leu93) was changed to alanine (L93A), and glutamic acid (Glu113) was changed to alanine (E113A) (Figure 6 and Figure S2B‐D). In addition, the phylogenetic tree displayed that the *Tp*flagellins shared a close genetic relationship with *Bs*flagellin (Figure S2A) and the three residues were highly conserved in TLR5‐activated flagellins, but not in TLR5 non‐activated flagellins (Figure 7A). All site‐directed mutant flagellins were prepared in recombinant form (Figure S2E). The IL‐6 and IL‐8 inducing activity of recombinant flagellins with these point mutations were shown in Figure 7B‐D. Strikingly, among the three single point mutants, *Tp*flagellins R89A significantly reduced the IL‐6 and IL‐8 levels, while *Tp*flagellins L93A and E113A just reduced a bit of IL‐6 and IL‐8 levels compared with the wild‐type flagellin. The double site‐specific mutants FlaB^R89A, E113A^ and FlaB^R89A, L93A^ were more attenuated in IL‐6 and IL‐8 transcriptions than FlaB^L93A, E113A^. The triple site‐specific mutant FlaB^R89A, L93A, E113A^ elicited far lower IL‐6 and IL‐8 transcriptions. Taken together, we concluded that the residue R89 in ND1 domain of *Tp*flagellins and its adjacent residues (*Tp*flagellins L93 and E113) constituted a hot spot for TLR5 activation. To determine whether the residues mutation affect the binding intensity of recombinant flagellins to the TLR5 ectodomain, co‐immunoprecipitation was used to detect the combination between site‐directed mutant flagellins and human TLR5. As shown in Figure 7E, these site‐directed mutations had varying degrees of impact on the binding efficiency of those flagellin molecules to TLR5.

## 4 DISCUSSION

We recently reported that purified *T pallidum* flagellins played an essential role in eliciting pro‐inflammatory cytokines in vitro.^43^ In the current study, we demonstrated that the ND1 domain of *Tp*flagellins binding to TLR5 receptor was required but not sufficient for TLR5 activation and IL‐6 and IL‐8 transcriptions. This finding gives insight into the pathogenesis of *T pallidum* and contributes to designing effective therapeutics and vaccines in the future.

Several studies have indicated that the release of inflammatory mediators caused by flagellins occurred through the interaction between flagellin and TLR5.^52^, ^53^, ^54^ However, as one of the smallest flagellin consisting only of the D0 and D1 domains, it was necessary to understand the TLR5 *Tp*flagellin interaction in more detail since the conflicts dated from different researchers in which some claimed that a central hypervariable domain was essential for activation of pro‐inflammatory gene expressions,^39^ others revealed that the conserved N‐terminal domain mediated the pro‐inflammatory response,^38^, ^40^ still other researcher indicated that the D0 domain of flagellin was required to produce a maximum TLR5‐mediated response.^42^ However, our data showed that the conserved D1 domain was responsible for inducing pro‐inflammatory response. This was consistent with the results of previous researchers.^21^, ^40^, ^55^ Moreover, in the experiment of mutant flagellins binding to TLR5, we found that although mutant flagellins △C and N lack CD1, which was shown to combine directly with TLR5 in a previous reported *Sd*Flg‐zfTLR5 structure^36^ and our *Tp*FlaB1‐hTLR5 complex (Figure 1E,F), they can still combine with TLR5. While mutant flagellins △N and C containing CD1 domain failed to combine with TLR5. This demonstrated that the ND1 domain of *Tp*flagellins played a major role in the primary binding of flagellin‐TLR5 complex. Meanwhile, the mutant flagellins △C and N containing ND1 domain failed to activate TLR5. This further indicated that although ND1 domain was involved in the primary binding, the △C and N may lack residues to guide receptor dimerization for TLR5 activation. Thus, the ND1 domain of *Tp*flagellins binding to TLR5 receptor was required but not sufficient for TLR5 activation.

*Tp*flagellins subdomain deletions revealed that the D1 domain played a key role in TLR5 activation, while the D0 domain has a minimal effect on TLR5 activation. This was the same as *St*flagellin,^40^ however, different from *Sd*flagellin.^36^ It may be related to the differences among species in the D0 domain. In addition, despite the phylogenetic tree (Figure S2A) revealed that *Tp*flagellins shared a close genetic relationship with *Bacillus subtilis* flagellin (*Bs*flagellin) and alignment through ClustalW showed that significant sequence homology existed in the conserved N‐ and C‐terminal between *Tp*flagellins and *Bs*flagellin (Figure 1A), the domains responsible for TLR5 activation were not quite the same. Compared with the D0 domain of *Bs*flagellin,^38^ D0 domain of *Tp*flagellins showed lower TLR5 activation. Although researchers suggested that the D0 domain was essential for TLR5 activation through the promotion of receptor dimerization, it was difficult to define the effect of C‐terminal D0 domain of flagellin in TLR5 activation because TLR5 in the *Sd*flagellin^D1‐D2^‐rTLR5^N14^ and *Bs*flagellin^cent^‐rTLR5^N14^ complex structures was derived from zebrafish and displayed only the N‐terminal region of zfTLR5, and the D0 domain was not located in the complex structure. Furthermore, a recent study indicated that flagellin was recognized by TLR5 through a more extensive interaction surface than crustal structure shown.^56^ Therefore, the key domains to guide receptor dimerization for TLR5 activation needed to be identified in the future.

Our mutational analyses of *Tp*flagellins‐mediated TLR5 activation displayed that R89 is especially critical for TLR5 signalling and its adjacent residues L93 and E113 seemed to play assistant roles for the interactions. Sequence analyses indicated that the three residues were highly conserved in TLR5‐activated flagellins, but not in TLR5 non‐activated flagellins. This was consistent with previous structural model of *Sd*flagellin^D1‐D2^‐rTLR5^N14^ and *Bs*flagellin^cent^‐rTLR5^N14^ complexes, in which TLR5 formed a cavity to interact with R89, L93, and E113.^38^ These site‐directed mutations not only had varying degrees of impact on TLR5 activation, but also affected the binding efficiency of those flagellin molecules to TLR5. This demonstrated that the three sites are essential for the combination of *Tp*flagellins and TLR5 in the primary binding site. Moreover, predicted 3D structures of *Tp*flagellins were similar to *Bs*flagellin in TLR5‐binding area (Figure 6). Taken together, we concluded that *Tp*flagellins R89 and its adjacent residues played a critical role for TLR5 activation in a conserved mechanism.

As a vaccine adjuvant, the potent antigenicity of flagellin might affect its potency and induce side effects, thus efforts should be made to reduce potential adverse effects induced by flagellin while maintaining its adjuvanticity. Many studies have proved that the middle hypervariable region of flagellin was replaced with the antigen, which could reduce the side effects. However, the fusion process may introduce inter‐molecular steric clashes or unexpected modifications, such as proteolysis or destabilization, which could reduce the efficacy of the flagellin‐antigen fusion vaccine. Moreover, the D2 and D3 domains could induce an unwanted toxic immune response. Therefore, it would be useful to identify the smallest naturally occurring flagellin containing only the D0 and D1 domains.

*T pallidum* flagellins contain only D0 and D1 domains and are one of the shortest flagellins. Although *T pallidum* flagellins lack the hypervariable domains, it exhibits similar TLR5‐binding affinity and TLR5 signalling activity to other flagellins. Flagellin, as the only TLR5‐specific agonist, has been developed as a vaccine adjuvant and the adjuvant activity of flagellin is mainly mediated by TLR5‐activating capacity.^57^ The induction of pro‐inflammatory cytokines and chemokines in a number of innate and non‐immune cell^33^, ^58^, ^59^, ^60^ mediated by MyD88‐dependent NF‐κB signalling is critical for the activation and development of antigen‐specific adaptive immune responses, which could promote cell‐mediated immunity via stimulation of Th1 cells and a marked recruitment in T and B lymphocytes to draining lymph nodes, and thus maximizes the chances of antigen‐specific lymphocytes encountering their cognate antigens.^61^ In addition, flagellin, a vaccine adjuvant, has the ability to induce macrophages to produce a variety of cytokines, which were central to the role of macrophages that mediate the transition from innate to adaptive immunity and enhance the microbicidal ability.^62^ For instance, IL‐6 secreted by macrophages could promote differentiation of B cells into plasma cells, activate cytotoxic T cells and lead to the recruitment of monocytes to the inflammation site^63^; IL‐8 is a potent chemoattractant for neutrophils, in which it also induces degranulation and morphological changes.^64^ Both of them could help to eliminate pathogens. CBLB502, as an anti‐radiation therapeutic, has been used to protect against gastrointestinal and haematopoietic acute radiation syndromes by removing the D2 and D3 domains from *sd*flagellin.^23^ However, given that CBLB502 protein seems to be unstable during purification and storage, and the D2 and D3 domains are not necessary for TLR5‐mediated immune response and could induce an unexpected adaptive immunity and cellular toxicity in the host; thus, the naturally *Tp*flagellins containing only the D0 and D1 domains are useful for the development of flagellin‐based anti‐pathogenic vaccines and anti‐radiation therapeutics.

All in all, our findings demonstrated that the ND1 domain of *Tp*flagellins binding to TLR5 is required but not sufficient for TLR5 activation and IL‐6 and IL‐8 transcriptions, which are consistent with the TLR5‐*Tp*flagellin complex structure analysis. Site‐directed mutational analyses revealed that at least three distinct sites (R89, L93 and E113) on flagellin were required in *Tp*flagellins‐mediated TLR5 activation. All of these will contribute to establishing the foundation work for the future design of *Tp*flagellins‐based syphilis vaccine.

## CONFLICT OF INTEREST

The authors declare no conflict of interest.

## AUTHOR’S CONTRIBUTIONS

Man Xu, Yafeng Xie and Yimou Wu conceived and designed the experiments. Man Xu, Manyi Tan, Kang Zheng and Yongjian Xiao performed the experiments. Man Xu, Feijun Zhao, Tiebing Zeng and Yimou Wu analysed the data. Man Xu, Yafeng Xie, Chuanhao Jiang and Yimou Wu wrote the paper. All authors read and approved the final manuscript.
