# Supplementary figures and images for: The N‐terminal D1 domain of Treponema pallidum flagellin binding to TLR5 is required but not sufficient in activation of TLR5
Source: J Cell Mol Med. 2019 Sep 7;23(11):7490–504. doi: 10.1111/jcmm.14617 (PMC6815820; doi:10.1111/jcmm.14617)

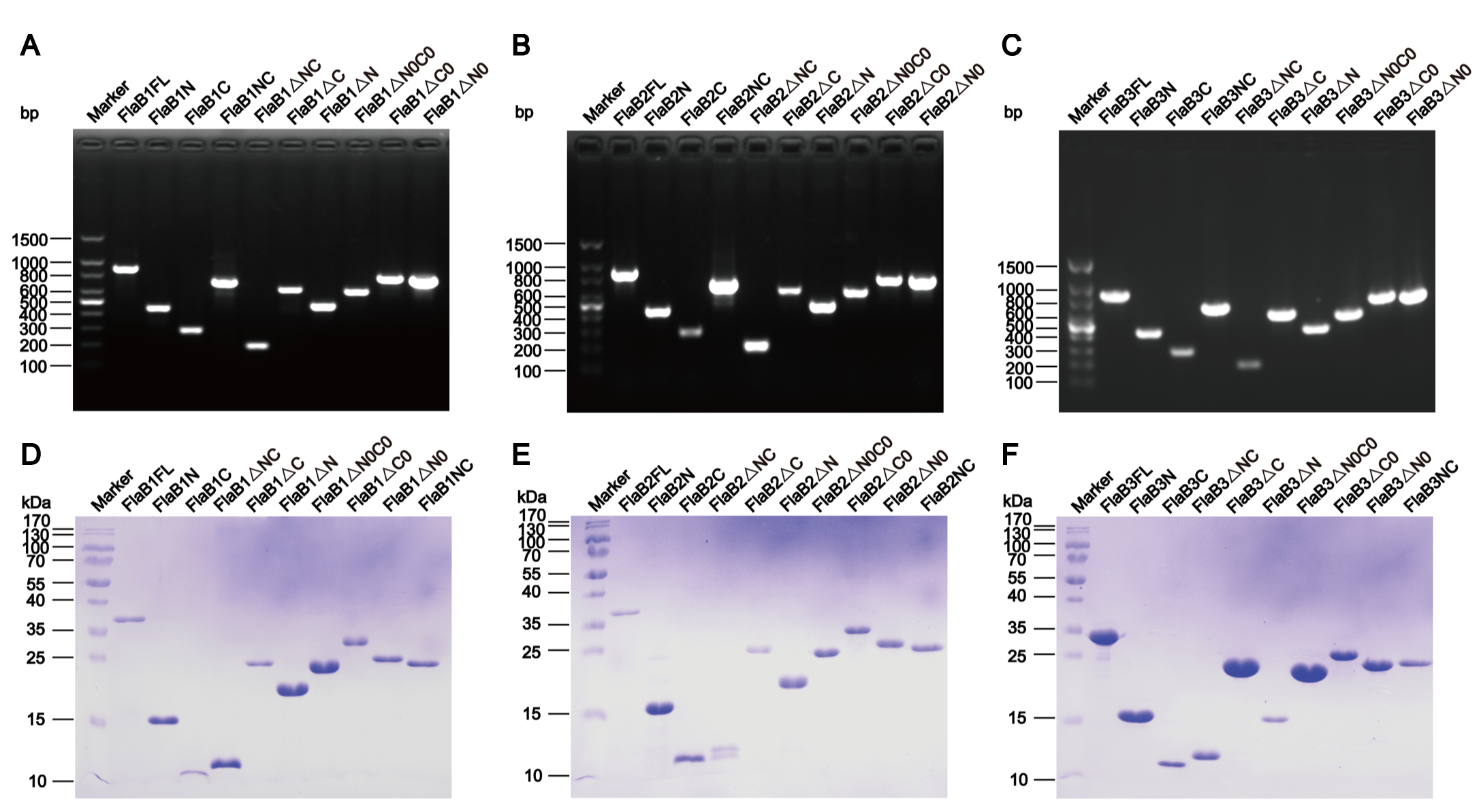

Supplement: Supplementary file 1 [file JCMM-23-7490-s001.tif]

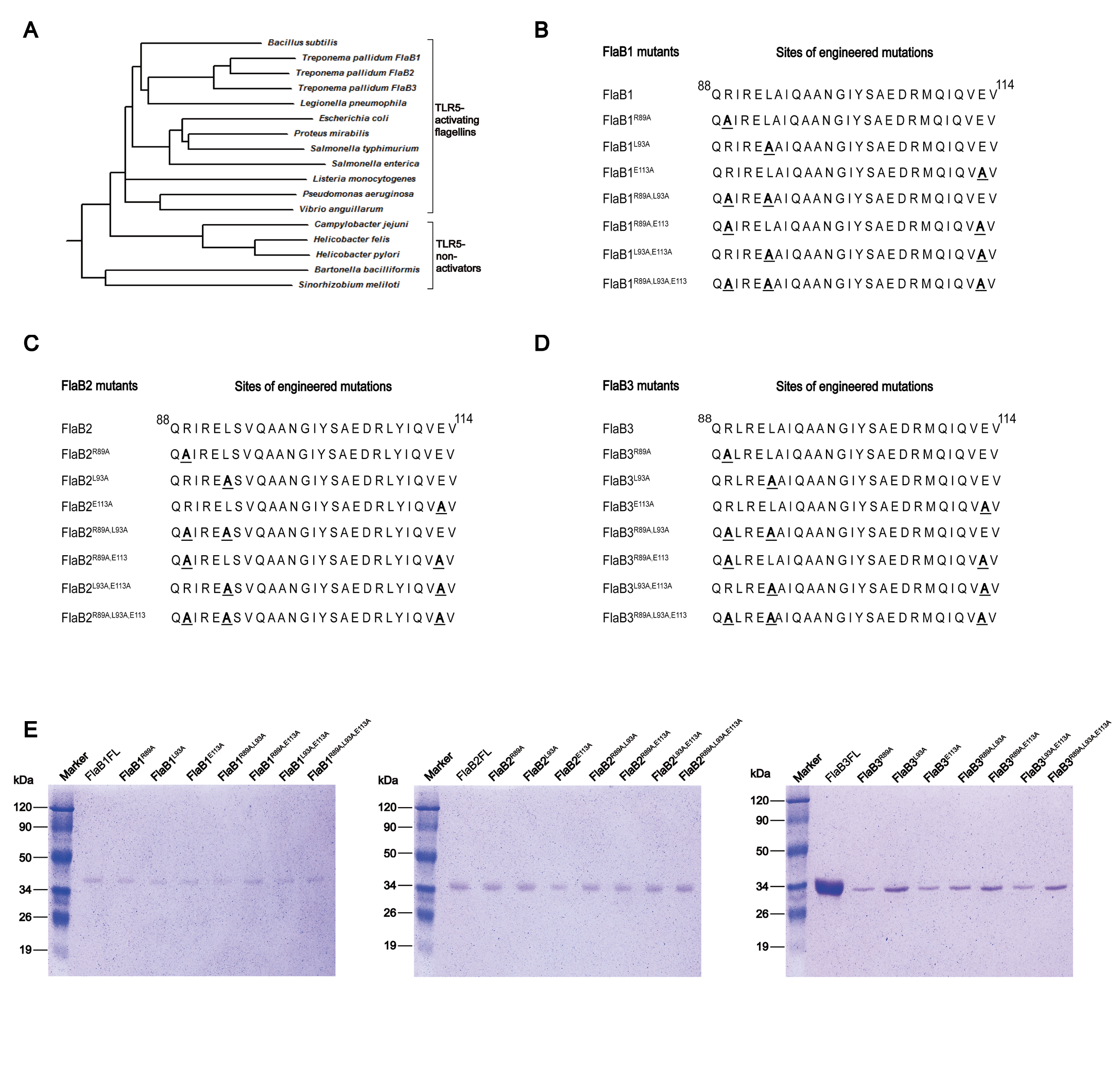

Supplement: Supplementary file 2 [file JCMM-23-7490-s002.tif]
