# Supplementary material for: The N‐terminal D1 domain of Treponema pallidum flagellin binding to TLR5 is required but not sufficient in activation of TLR5
Source: J Cell Mol Med. 2019 Sep 7;23(11):7490–504. doi: 10.1111/jcmm.14617 (PMC6815820; doi:10.1111/jcmm.14617)
Supplement: Supplementary file 3 [file JCMM-23-7490-s003.docx]

**Table s1 Primer sequences used for cloning of fragments**

| Gene | Primer Sequences(5’ to 3’) | Restriction enzyme | Amplicon  size (bp) |
| --- | --- | --- | --- |
| FlaB1N | F:CGC**GGATCC**ATGATTATCAATCACAAC | *Bam*HI | 426 |
|  | R: CCG**CTCGAG**TTAGGAGAAGCGGCCCGTGAG | *Xho*I |  |
| FlaB1C | F:CGC**GGATCC**ATGATCGGCACCATCGATGCTG | *Bam*HI | 267 |
|  | R:CCG**CTCGAG**TTACCGGAGAATTGAGAG | *Xho*I |  |
| FlaB1△N0 | F: CGC**GGATCC**ATGCGCAGCCAAATCCGCG | *Bam*HI | 705 |
|  | R: CCG**CTCGAG**TTACCGGAGAATTGAGAG | *Xho*I |  |
| FlaB1△C0 | F: CGC**GGATCC**ATGATTATCAATCACAAC | *Bam*HI | 729 |
|  | R: CCG**CTCGAG**TTACTCAGCTGCCTGCAGGT | *Xho*I |  |
| FlaB1△N0C0 | F: CGC**GGATCC**ATGCGCAGCCAAATCCGCG | *Bam*HI | 576 |
|  | R: CCG**CTCGAG**TTACTCAGCTGCCTGCAGGT | *Xho*I |  |
| FlaB1△N | F: CGC**GGATCC**ATGCGCACTGAAGGTGAGAACG | *Bam*HI | 444 |
|  | R: CCG**CTCGAG**TTACCGGAGAATTGAGAG | *Xho*I |  |
| FlaB1△C | F: CGC**GGATCC**ATGATTATCAATCACAAC | *Bam*HI | 606 |
|  | R: CCG**CTCGAG**TTAGCTCTTGTTGGCCGAGTCT | *Xho*I |  |
| FlaB1△NC | F: CGC**GGATCC**ATGCGCACTGAAGGTGAGAACG | *Bam*HI | 180 |
|  | R: CCG**CTCGAG**TTAGCTCTTGTTGGCCGAGTCT | *Xho*I |  |
| FlaB1NC | F1:AGCATCGATGGTGCCGATGGAGAAGCGGCCCGTGAG |  |  |
|  | R1:CTCACGGGCCGCTTCTCCATCGGCACCATCGATGCT |  |  |
|  | F2: CGC**GGATCC**ATGATTATCAATCACAAC | *Bam*HI | 693 |
|  | R2: CCG**CTCGAG**TTACCGGAGAATTGAGAG | *Xho*I |  |
| FlaB2N | F: CGC**GGATCC**ATGATCATCAATCACAACATG | *Bam*HI | 426 |
|  | R: CCC**AAGCTT**CTACGCGAAGCGACCAGTGAG | *Hin*dIII |  |
| FlaB2C | F:CGC**GGATCC**ATGATCGGTACGCTTGATCAG | *Bam*HI | 267 |
|  | R:CCG**CTCGAG**CTAACGCAAGAGGCTTAGAAC | *Xho*I |  |
| FlaB2△N0 | F: CGC**GGATCC**ATGCGGAGCCAGATCCGTGGT | *Bam*HI | 705 |
|  | R: CCG**CTCGAG**CTAACGCAAGAGGCTTAGAAC | *Xho*I |  |
| FlaB2△C0 | F: CGC**GGATCC**ATGATCATCAATCACAACATG | *Bam*HI | 729 |
|  | R: CCC**AAGCTT**CTACTCAGCTGCCTGCAAGTT | *Hin*dIII |  |
| FlaB2△N0C0 | F: CGC**GGATCC**ATGCGGAGCCAGATCCGTGGT | *Bam*HI | 576 |
|  | R: CCC**AAGCTT**CTACTCAGCTGCCTGCAAGTT | *Hin*dIII |  |
| FlaB2△N | F: CGC**GGATCC**ATGCGTCAAGGCGGGGAGAAC | *Bam*HI | 444 |
|  | R: CCG**CTCGAG**CTAACGCAAGAGGCTTAGAAC | *Xho*I |  |
| FlaB2△C | F: CGC**GGATCC**ATGATCATCAATCACAACATG | *Bam*HI | 606 |
|  | R: CCC**AAGCTT**CTATGCGCGGTTGGCCTTTTC | *Hin*dIII |  |
| FlaB2△NC | F:CGC**GGATCC**ATGCGTCAAGGCGGGGAGAAC | *Bam*HI | 180 |
|  | R:CCC**AAGCTT**CTATGCGCGGTTGGCCTTTTC | *Hin*dIII |  |
| FlaB2NC | F1:CTGATCAAGCGTACCGATCGCGAAGCGACCAGTGAG |  |  |
|  | R1:CTCACTGGTCGCTTCGCGATCGGTACGCTTGATCAG |  |  |
|  | F2: CGC**GGATCC**ATGATCATCAATCACAACATG | *Bam*HI | 693 |
|  | R2: CCG**CTCGAG**CTAACGCAAGAGGCTTAGAAC | *Xho*I |  |
| FlaB3N | F:CGC**GGATCC**ATGATTATCAATCACAACATG | *Bam*HI | 426 |
|  | R:CCG**GAATTC**TTAGGAGAAGCGGCCCGTGAG | *Eco*RI |  |
| FlaB3C | F:CGC**GGATCC**ATGATCGGTACGCTTGATAGC | *Bam*HI | 267 |
|  | R:CCG**GAATTC**TTACTGCATCAAGCGGAG | *Eco*RI |  |
| FlaB3△N0 | F: CGC**GGATCC**ATGCGTAGCCAAATCCGCGGC | *Bam*HI | 705 |
|  | R: CCG**GAATTC**TTACTGCATCAAGCGGAG | *Eco*RI |  |
| FlaB3△C0 | F: CGC**GGATCC**ATGATTATCAATCACAACATG | *Bam*HI | 729 |
|  | R: CCG**GAATTC**TTACTCGGCTGCCTGCAGATT | *Eco*RI |  |
| FlaB3△N0C0 | F: CGC**GGATCC**ATGCGTAGCCAAATCCGCGGC | *Bam*HI | 576 |
|  | R: CCG**GAATTC**TTACTCGGCTGCCTGCAGATT | *Eco*RI |  |
| FlaB3△N | F: CGC**GGATCC**ATGCGCGAGTCTGCCCTTGGG | *Bam*HI | 444 |
|  | R: CCG**GAATTC**TTACTGCATCAAGCGGAG | *Eco*RI |  |
| FlaB3△C | F: CGC**GGATCC**ATGATTATCAATCACAACATG | *Bam*HI | 606 |
|  | R: CCG**GAATTC**TTAGACCTTGTTCGCCCCGTC | *Eco*RI |  |
| FlaB3△NC | F:CGC**GGATCC**ATGCGCGAGTCTGCCCTTGGG | *Bam*HI | 180 |
|  | R:CCG**GAATTC**TTAGACCTTGTTCGCCCCGTC | *Eco*RI |  |
| FlaB3NC | F1:GCTATCAAGCGTACCGATGGAGAAGCGGCCCGTGAG |  |  |
|  | R1:CTCACGGGCCGCTTCTCCATCGGTACGCTTGATAGC |  |  |
|  | F2:CGC**GGATCC**ATGATTATCAATCACAACATG | *Bam*HI | 693 |
|  | R2: CCG**GAATTC**TTACTGCATCAAGCGGAG | *Eco*RI |  |
