# Supplementary material for: The N‐terminal D1 domain of Treponema pallidum flagellin binding to TLR5 is required but not sufficient in activation of TLR5
Source: J Cell Mol Med. 2019 Sep 7;23(11):7490–504. doi: 10.1111/jcmm.14617 (PMC6815820; doi:10.1111/jcmm.14617)
Supplement: Supplementary file 4 [file JCMM-23-7490-s004.docx]

**Table s2 Primer sequences used for cloning of the point mutation gene**

| Gene | Template | Sequence(5’ to 3’) |
| --- | --- | --- |
| FlaB1*^R89A^* | *FlaB1* | 5’-CCGACATCATGCAG**GC**TATCCGAGAGCTTG-3’ |
| FlaB1*^L93A^* | *FlaB1* | 5’-AGCGTATCCGAGAG**GC**TGCAATTCAAGCGG-3’ |
| FlaB1*^E113A^* | *FlaB1* | 5’-TGCAGATCCAGGTGG**C**AGTTTCGCAGCTTG-3’ |
| FlaB1 *^R89A, L93A^* | FlaB1*^R89A^* | 5’-AGGCTATCCGAGAG**GC**TGCAATTCAAGCGG-3’ |
| FlaB1 *^R89, E113A^* | FlaB1*^E114A^* | 5’-CCGACATCATGCAG**GC**TATCCGAGAGCTTG-3’ |
| FlaB1 *^L93A, E113A^* | FlaB1*^E114A^* | 5’-AGCGTATCCGAGAG**GC**TGCAATTCAAGCGG-3’ |
| FlaB1 *^R89A, L93A, E113A^* | FlaB1 *^R89A, L93A^* | 5’-TGCAGATCCAGGTGG**C**AGTTTCGCAGCTTG-3’ |
| FlaB2*^R89A^* | *FlaB2* | 5’-CCGACGTTATCCAG**GC**CATTCGCGAACTGA-3’ |
| FlaB2*^L93A^* | *FlaB2* | 5’-AGCGCATTCGCGAA**GC**GAGTGTGCAGGCGG-3’ |
| FlaB2*^E113A^* | *FlaB2* | 5’-TCTACATTCAGGTAG**C**GGTCTCTCAGTTGG-3’ |
| FlaB2 *^R89A, L93A^* | FlaB2*^R89A^* | 5’- AGGCCATTCGCGAA**GC**GAGTGTGCAGGCGG-3’ |
| FlaB2 *^R89, E113A^* | FlaB2*^E114A^* | 5’-CCGACGTTATCCAG**GC**CATTCGCGAACTGA-3’ |
| FlaB2*^L93A, E113A^* | FlaB2*^E114A^* | 5’-AGCGCATTCGCGAA**GC**GAGTGTGCAGGCGG-3’ |
| FlaB2 *^R89A, L93A, E113A^* | FlaB2 *^R89A, L93A^* | 5’-TCTACATTCAGGTAG**C**GGTCTCTCAGTTGG-3’ |
| FlaB3*^R89A^* | *FlaB3* | 5’-CTGAAATCGTCCAG**GC**CCTGAGGGAGCTTG-3’ |
| FlaB3*^L93A^* | *FlaB3* | 5’-AGCGCCTGAGGGAG**GC**TGCAATCCAGGCGG-3’ |
| FlaB3*^E113A^* | *FlaB3* | 5’-TGCAGATCCAGGTGG**C**AGTTTCACAGCTTG-3’ |
| FlaB3 *^R89A, L93A^* | FlaB3*^R89A^* | 5’- AGGCCCTGAGGGAG**GC**TGCAATCCAGGCGG-3’ |
| FlaB3 *^R89, E113A^* | FlaB3*^E114A^* | 5’-CTGAAATCGTCCAG**GC**CCTGAGGGAGCTTG-3’ |
| FlaB3 *^L93A, E113A^* | FlaB3*^E114A^* | 5’-AGCGCCTGAGGGAG**GC**TGCAATCCAGGCGG-3’ |
| FlaB3 *^R89A, L93A, E113A^* | FlaB3 *^R89A, L93A^* | 5’-TGCAGATCCAGGTGG**C**AGTTTCACAGCTTG-3’ |
